# Exploring Variation in Ovine *KRTAP19-5* and Its Effect on Fine Wool Fibre Curvature in Chinese Tan Sheep

**DOI:** 10.3390/ani14152155

**Published:** 2024-07-24

**Authors:** Lingrong Bai, Huitong Zhou, Wenhao Li, Jinzhong Tao, Jon G. H. Hickford

**Affiliations:** 1 International Wool Research Institute, Faculty of Animal Science and Technology, Gansu Agricultural University, Lanzhou 730070, China; lingrong.bai@lincolnuni.ac.nz (L.B.); huitong.zhou@lincoln.ac.nz (H.Z.); 2 Gene-Marker Laboratory, Faculty of Agriculture and Life Sciences, Lincoln University, Lincoln 7647, New Zealand; 3 Plateau Livestock Genetic Resources Protection and Innovative Utilization Key Laboratory of Qinghai Province, Key Laboratory of Animal Genetics and Breeding on Tibetan Plateau, Ministry of Agriculture and Rural Affairs, Qinghai Academy of Animal Science and Veterinary Medicine, Qinghai University, Xining 810016, China; qhdxlwh@163.com; 4 College of Animal Science and Technology, Ningxia University, Yinchuan 750021, China; tao_jz@nxu.edu.cn

**Keywords:** keratin-associated protein, KAP19-5, variation, wool traits, fine wool, heterotypic hair fibre, Chinese Tan sheep

## Abstract

**Simple Summary:**

Wool is a natural fibre with unique biological, chemical and physical properties, yet it struggles with issues related to having inconsistent quality. Improving the consistency of wool traits through selective breeding is hindered by our limited understanding of the mechanisms, genetic or otherwise underlying fibre variability. To improve understanding, this study focuses on characterisation of the *KRTAP19-5* gene that would encode a high glycine and tyrosine containing keratin-associated protein. The study reveals variation within the gene and how that appears to influence the mean fibre curvature of the fine wool fibres from Chinese Tan sheep.

**Abstract:**

Sheep’s wool is known to have unique biological, physical and chemical properties. The fibre primarily consists of proteins, but these have amino acid sequence variation, and at the phenotypic level wool fibre varies considerably. This can affect its utility and value. Unravelling the genetic factors that underpin the protein and phenotypic variability is crucial if we are to contemplate improving wool quality. Accordingly, this study investigates the high glycine and tyrosine content keratin-associated protein 19-5 gene (*KRTAP19-5*) in sheep. PCR-single strand confirmation polymorphism analysis, coupled with DNA sequencing of a region spanning whole coding sequence, revealed six sequence variants containing seven single nucleotide polymorphisms (SNPs). Five of the SNPs were located within the coding region, with four leading to amino acid changes if expressed. In 247 Chinese Tan sheep derived from 10 sire-lines, and renowned for their distinct ‘spring-like’ crimped wool at up to approximately 35 days after birth, one of the variants was found to be associated with decreased curvature of the fine wool fibres in the fleece. No associations were detected with other fibre traits or with variation in the heterotypic hair fibres of the Tan sheep. While these findings may be useful for developing gene markers to alter mean wool fibre curvature and improve sheep breeding, many other genes and environmental factors are known to contribute to variation in fibre traits.

## 1. Introduction

Wool is a natural fibre renowned for possessing unique properties [1], but as a natural fibre it can be is quite variable. This can restrict its use, and thus it influences its value. Wool fibre is primarily composed of two types of protein, the wool keratins and the keratin-associated proteins (KAPs) [2]. Understanding the genes regulating the production of these proteins is essential to improving our understanding of wool variability and thus the quality of wool.

The keratin-associated proteins have a diverse composition and comprise numerous individual genes and proteins [2,3]. They can be broadly categorised into three groups based on their amino acid composition: the high sulphur (HS) proteins, the ultra-high sulphur (UHS) proteins and the high glycine and tyrosine (HGT) proteins [4]. While the exact number of ovine KAP genes (designated *KRTAPs*) is unknown, studies in humans reveal 89 *KRTAPs*. These have been assigned into 12 HS-KAP families (containing 25 *KRTAPs*), 6 UHS-KAP families (containing 47 *KRTAPs*) and 7 HGT-KAP families (containing 17 *KRTAPs*) [5,6,7].

The quantity of HGT-KAPs present in wool fibres varies, from under 1% in Lincoln sheep wool, to 4–12% in Merino sheep wool [8]. The HGT-KAPs are found at a greater abundance in the orthocortex of the wool fibre, compared to paracortex, and a decrease in HGT-KAP content appears to be at least partially responsible for the felting lustre mutant observed in Merino sheep [9]. The identification of five additional HGT-KAP genes in sheep, which are not found in humans [10,11,12,13], suggests the ovine HGT-KAP family may be more diverse than in humans. Together, the evidence suggests that HGT-KAPs may play a role in regulating wool characteristics.

Gene members of all the known HGT-KAP families except for the KAP19 family have been identified and investigated in sheep [14]. In humans, KAP19 is acknowledged to be the largest HGT-KAP family, and it consists of seven genes [15], thus it is surprising that none of the *KRTAP19* genes has been investigated in sheep. While genome constructs and databases identify KAP genes in other animals including alpaca, llama, cattle, yaks, dogs and goats, humans [15] are the only species where the KAP19 family has been characterised.

The Tan sheep, a breed indigenous to China, is renowned for producing wool with a distinctive ‘spring-like’ crimp, especially up to approximately 35 days after birth, in what is traditionally referred to as ‘Er-mao’ [16]. In this setting, this study sought to identify ovine *KRTAP19-5*, ascertain whether the gene was variable, and investigate whether variation if detected, is associated with wool traits in Chinese Tan sheep.

## 2. Materials and Methods

### 2.1. Sheep Investigated and Wool Trait Measurement

Two separate groups of sheep were investigated in this study. The first group compromised 68 sheep selected from various farms. They were chosen so as to represent unrelated individuals of differing breed, which have been selected historically for meat, wool and dual-purpose production system, and so as to create a diverse base to ascertain the likely extent of DNA sequence variation in *KRTAP19-5*. This group was solely used for screening for variation in ovine *KRTAP19-5*, and was not subjected to association analyses, given that wool samples had not been collected, and neither had wool trait information.

The second group comprised 247 Chinese Tan lambs derived from ten sire-lines, and these were chosen to enable the relationship between variation in *KRTAP19-5* variation and variation in selected fibre traits to be tested. Most of the lambs were born as singles, but six of them (three pairs) were born as twins. To prevent potential confounding effects, the twins were excluded from the analyses, leaving 241 single lambs for the association study.

Wool samples were collected from the mid-side region of the Chinese Tan lambs at Er-mao (day 35 post-partum). For each sample, fine wool fibres and heterotypic hair fibres were manually separated from each other based on the noticeable difference in fibre diameter and length. This separation was achieved using a flannel board that the wool samples could be spread out on. Using a rigid card to press the base of all the fibres against the board, the typically longer and higher fibre diameter heterotypic hair fibres could be pulled out of the wool sample. This process was repeated to ensure all the heterotypic hair fibres were isolated from the fine wool fibres.

The fine and heterotypic fibres from each sample were then measured for mean fibre diameter (MFD), fibre diameter standard deviation (FDSD), coefficient of variation of fibre diameter (CVFD) and mean fibre curvature (MFC). The measurements on the heterotypic fibres were undertaken by the New Zealand Wool Testing Authority, Napier, New Zealand using International Wool Textile Organisation endorsed tests, while the measurement of the fine fibres was undertaken by Pastoral Measurements Limited (Timaru, New Zealand). 

Blood samples from each sheep were collected onto TFN paper (Munktell Filter AB, Sweden) and the cards dried for storage. For analysis of *KRTAP19-5*, punches of 1.2 mm in diameter were taken from the blood spot on the TFN paper, and DNA for PCR amplification (which has attached to the TFN paper), was prepared using a two-step washing process [17]. This method involved incubating the punches in 20 mM NaOH solution for 30 min at room temperature, removal of the NaOH and a subsequent single wash with 1× TE^−1^ buffer (10 mM Tris-HCl, 0.1 mM EDTA, pH 8.0).

### 2.2. PCR Amplification and Single Strand Conformation Analysis

A pair of PCR primers were designed to amplify ovine *KRTAP19-5* based on the *KRTAP19-5* sequence ENSOARG00020032146 (Ensembl, *KRTAP19-5* sequence derived from chromosome 1 of the ovine genome assembly ARS-UI_Ramb_v2.0:CM028704.1; 3 February 2021). The sequences of these primers were 5′-TCTGACCAGCAGCAGCAGC-3′ (forward primer) and 5′-ATCTTGGCCTTAATCTTAGAC-3′ (reverse primer), and they were synthesised by Integrated DNA Technologies (Coralville, IA, USA). The PCR amplifications were conducted in 15-μL reactions. These contained the cleansed genomic DNA on a single punch of the TFN paper, 150 μM of each deoxynucleotide triphosphate (dNTP; Bioline, London, UK), 0.25 μM of each primer, 2.5 mM Mg^2+^, 0.5 U of Taq DNA polymerase (Qiagen, Hilden, Germany), and 1× the reaction buffer supplied with the enzyme. The thermal profile for amplification included an initial denaturation step for 2 min at 94 °C, followed by 35 cycles of 30 s at 94 °C, 30 s at 58 °C and 30 s at 72 °C, and with a final extension step for 5 min at 72 °C. The thermal cycling was carried out in S1000 thermal cyclers (Bio-Rad, Hercules, CA, USA). A positive control (sheep genomic DNA) and negative control (no DNA) were also run with the amplification reactions.

The PCR amplicons produced were analysed using a single strand conformation polymorphism (SSCP) approach. For this, a 0.7-μL aliquot of each amplicon was mixed with 7 μL of gel loading dye (0.025% bromophenol blue, 0.025% xylene-cyanol, 98% formamide, and 10 mM EDTA). After denaturation of the double-stranded DNA at 95 °C for 5 min, the samples were cooled on wet ice and loaded on 16 cm × 18 cm, 10% acrylamide: bisacrylamide (37.5:1) (Bio-Rad) gels that contain 5% glycerol. Electrophoresis of the samples was conducted at 390 volts and 7 °C in Protean II xi cells (Bio-Rad), for 19 h using 0.5× TBE run buffer. Upon completion of the electrophoresis, the SSCP gels were fixed and stained in a solution containing 10% ethanol, 0.5% acetic acid, and 0.2% silver nitrate for 10 min. The gels were rinsed once with distilled water and then developed with a solution of 3% NaOH and 0.1% HCOH until dark staining bands appeared on the yellow background. At that point, development was stopped by removing the developing solution and addition of a solution containing 10% ethanol and 0.5% acetic acid. With the PCR-SSCP approach used, different variant sequences of *KRTAP19-5* were expected to produce different banding patterns on the gels, with each DNA sequence producing two bands that correspond to the two strands of the DAN for any given variant. Homozygous sheep are therefore expected to produce two bands and heterozygous sheep four bands.

### 2.3. DNA Sequencing and Sequence Analysis

PCR amplicons displaying different SSCP banding patterns (or sequence variants) from sheep seemingly homozygous for the gene region amplified, were directly sequenced in both directions at the Lincoln University DNA sequencing facility (Lincoln University, Lincoln, New Zealand). Typically three sheep per homozygous variant, if available, were chosen for sequencing, and with sequencing being undertaken in both directions on an Applied Biosystems Model 3500XL Genetic Analyzer (Waltham, MA, USA) using POP7 polymer and a 24 capillary array. The raw sequence reads were aligned using DNAMAN XL (version 10, Lynnon BioSoft, Vaudreuil, QC, Canada). Individual nucleotide calls are expected to match across all six raw reads using this approach. Variants that were only detected in heterozygous sheep underwent sequencing using a gel separation-based sequencing approach [18]. In this approach, a gel slice corresponding to a SSCP band of the variant was excised form the polyacrylamide gel, macerated, and then used as a template for re-amplification with the original primers. This resulting second amplicon, was checked to insure it produced the expected ‘homozygous’ pattern using the PCR-SSCP gel system, then sequenced directly as above for the homozygous sheep. Once again, this approach was used with three separate sheep and each variant isolated using this approach was sequenced in triplicate in both directions. Once final sequences had been obtained, the open reading frame was determined and translation undertaken using DNAMAN XL. The finalized sequences were then subjected to a phylogenetic analysis, using the same software.

### 2.4. Statistical Analyses

Statistical analyses were conducted using Minitab version 16 (Minitab Inc., State College, PA, USA). General linear models (GLMs) were used to assess the impact of the presence or absence of the ovine *KRTAP19-5* variants on the various wool traits that were measured. To determine whether sire or gender would be included in the models, a univariate Pearson Chi-square test was performed to explore the association between these variables and the measure wool traits. Sire was identified to have an influence on all the wool traits measured and hence was included as an explanatory factor in all the models, while gender was identified as a factor affecting some of the wool traits. The linear model was:

Y_jkl_ = µ + V_j_ + G_k_ + S_l_ + e_jkl_

where Y_jkl_ is the phenotypic value for the _jkl_th sheep, µ is the group raw mean for the trait, V_j_ is the effect of the _j_th variant (presence and absence), G_k_ is the effect of gender, S_l_ is the effect of the lth sire, and e_jkl_ is the random residual effect. Significance was accepted at *p* < 0.05.

## 3. Results

**Figure 1:**
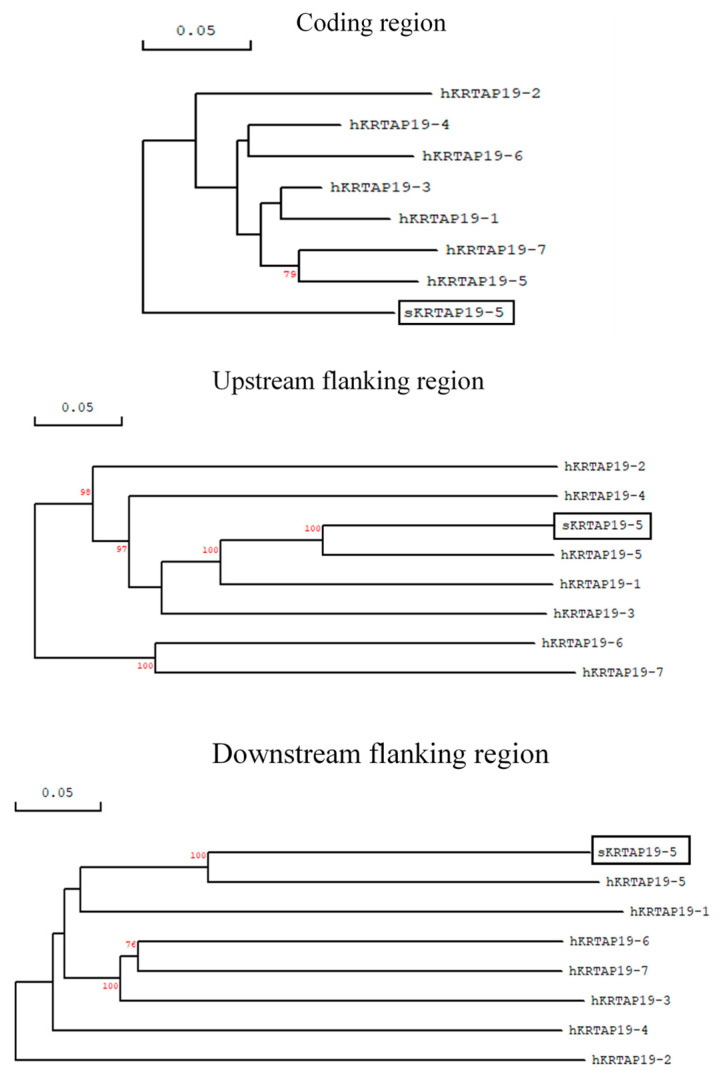
Phylogenetic analyses of sheep *KRTAP19-5* with known gene members of the human KAP19 family. The sheep *KRTAP19-5* sequence is labelled with the prefix “s” and highlighted in a box, while human genes are denoted by prefix “h”. Analyses are conducted for three different regions: the coding region, as well as the 1-kb upstream and downstream flaking regions. Bootstrap confidence values are indicated at the forks, with only values over 70% shown. Scale bars represent a rate of 0.05 nucleotide substitution per site.

The ovine *KRTAP19-5* sequence derived from the ovine genome assembly ARS-UI_Ramb_v2.0:CM028704.1, and as presented in Ensembl with the identification number ENSOARG00020032146, is an intronless gene with a coding sequence that spans 222 base pairs. Despite sharing 72.3–80.8% sequence homology with human *KRTAP19-n* sequences, this coding sequence does not exhibit a close relationship with any specific human *KRTAP19* genes. However, the upstream and downstream flanking sequences of the coding sequence have close identity to human *KRTAP19-5* (Figure 1). This suggests that the Ensembl-identified ovine *KRTAP19-5* sequence represents the ortholog of human *KRTAP19-5*.

The ovine *KRTAP19-5* sequence ENSOARG00020032146 also exhibits similarities to expressed sequence tags obtained from sheep wool follicles or skin (Supplementary Table S1), which suggests that the gene is expressed.

**Figure 2:**
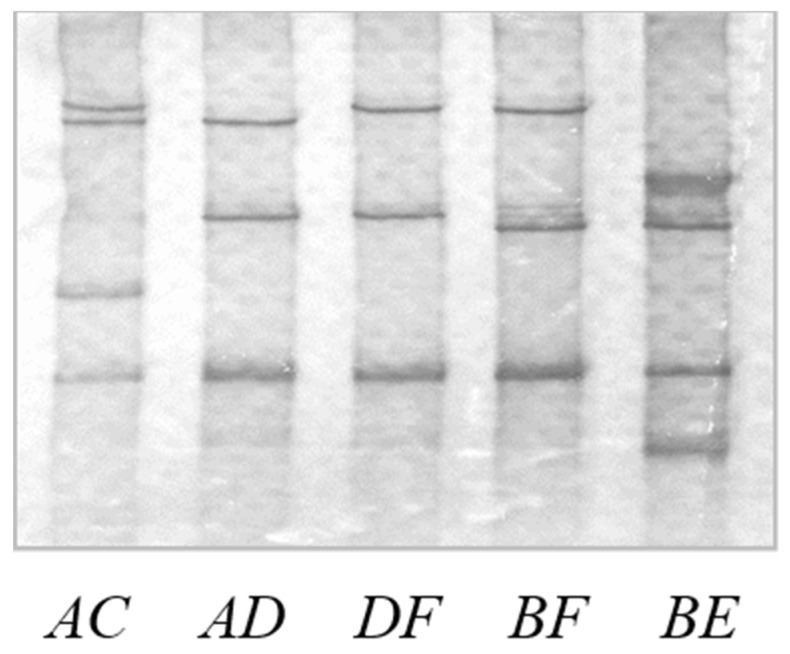
PCR-SSCP patterns of ovine *KRTAP19-5*. Six different banding patterns (*A* to *F*), corresponding to six different DNA haplotypes are observed in heterozygous forms. Different variant sequences of *KRTAP19-5* are expected to produce different banding patterns on the gels, with each DNA sequence producing two bands that correspond to the two strands of the DAN for any given variant.

**Figure 3:**
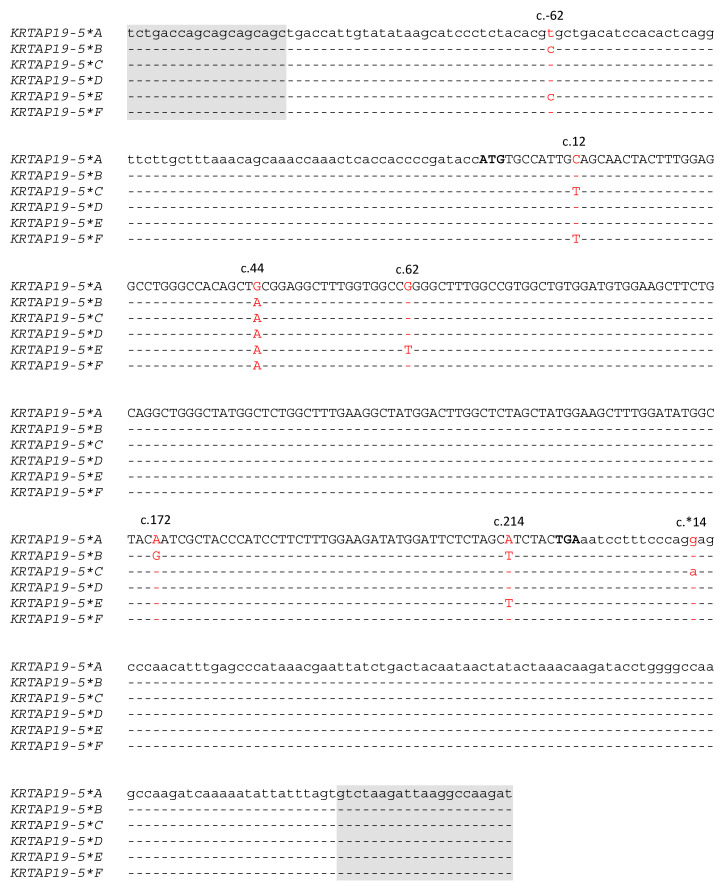
Alignment of the six ovine *KRTAP19-5* variant sequences using DNAMAN XL. The positions of seven SNPs identified among the six variant sequences (*A* to *F*) are indicated, with nucleotides shown in red. Nucleotides within the coding region are shown in upper case, while those outside the coding region are in lower case. The start codon and stop codon are shown in bold. Dashes represent nucleotide sequences identical to the top sequence. The primer binding regions are shaded.

The PCR-SSCP analysis of ovine *KRTAP19-5*, including 112 bp upstream and 132 bp downstream of the intronless coding sequence, revealed six distinct banding patterns (Figure 2), which upon corresponded to six unique, but closely-related sequence variants or haplotypes (Figure 3). A total of seven single nucleotide polymorphisms (SNPs) were identified, with five located in the coding region. Among the SNPs in the exons, four were non-synonymous (c.44G/A, c.62G/T, c.172A/G and c.214A/T) and would result in the amino acid substitutions p.Tyr15Cys, p.Arg21Leu, p.Asn58Asp and p.Ile72Phe respectively. The DNA sequence of variant *F* was identical to ENSOARG00020032146.

In the 68 sheep used for variation screening, the variant frequencies observed were 19.4%, 41.9%, 10.5%, 19.4%, 5.6%, and 3.2% for variants *A* to *F*, respectively.

In the 241 Chinese Tan sheep used for the association study, all the variants (*A* to *F*) were detected, with frequencies of 34.0%, 18.5%, 27.2%, 8.7%, 3.3% and 8.3% respectively. Variant *E* was detected at a frequency below 5% and was therefore of insufficient sample size to subsequently include in the association analyses, because the sheep carrying that variant could potentially bias the outcome of the models.

The variation detected in *KRTAP19-5* was associated with variation in MFC for the fine wool fibres of the Tan sheep (Table 1), with the presence of variant *B* being associated with decreased MFC (absent 65.3 ± 1.19°/mm vs. present 62.3 ± 1.45°/mm, *p* = 0.039). However, no associations were observed for other fibre traits, or with the heterotypic hair fibres.

## 4. Discussion

This study describes the identification of a member of the ovine KAP19 family. While resolving its identity solely based on its coding sequence is difficult, the high similarity of the upstream and downstream sequences flanking that coding sequence to the human genome, suggests it is likely an orthologue to human *KRTAP19-5*. This also suggests that unexplained evolutionary processes may have occurred in producing the human and sheep loci. In this respect, differences in evolutionary patterns between the coding region and flanking regions has been previously observed in the *KRTAP1* gene family [19], likely driven by distinct evolutionary forces. The evolution of the upstream and downstream regions of genes ensures beneficial regulation and expression of a gene, whereas the coding region evolves to maintain the preferred structure and function of the expressed protein.

Bioinformatics analyses of *KRTAP* coding sequences across 22 mammalian species suggests that HS-, UHS- and HGT-*KRTAPs* may have followed distinct evolutionary paths [20]. For example, the HS- and UHS-*KRTAPs* exhibit high rates of concerted evolution, likely driven by gene conversion and recombination events [20], with the *KRTAP1* family serving as a well-studied example [19,21]. In contrast, the HGT-*KRTAPs* display a more dynamic evolutionary pattern, characterised by signatures of positive selection and less evidence of gene conversion or recombination [20]. Given that *KRTAP19* belongs to the HGT group, future characterisation of additional *KRTAP19* family members in sheep and sequence analyses of these family members may provide further information into the evolution of the *KRTAP19* gene family and the HGT-*KRTAPs*.

A distinction also seems to arise between the sheep and human orthologs regarding the location of expression of *KRTAP19-5*. In sheep, *KRTAP19-5* appears to be expressed in wool follicles, as evidenced by the isolation of ESTs bearing similarities to the *KRTAP19-5* sequence from wool follicles or skin. In contrast, the expression of *KRTAP19-5* was not detected in human hair follicles by either cDNA library screening or in-situ hybridization studies [15].

The identification of six sequence variants in the region amplified of ovine *KRTAP19-5* suggests a high level of sequence diversity. The detection of seven SNP results in a calculated SNP density of 17.2 SNPs/kb in the region amplified, excluding the primer binding sequences. This SNP density is comparable to the densities found in *KRTAP1-2* [22], *KRTAP8-1* [23], *KRTAP28-1* [24] and *KRTAP36-2* [13], but less than the densities found in *KRTAP1-3* [25], *KRTAP1-4* [26], *KRTAP2-1* [27] and *KRTAP20-1* [28]. For context, the average SNP density in the sheep genome has been cited to be approximately 4.9 SNPs/kb [29], but our findings suggest that ovine *KRTAP19-5* has nucleotide variation at close to four times that rate. The non-synonymous SNPs identified in ovine *KRTAP19-5* may induce structural alterations in the protein, whereas synonymous SNPs and those outside the coding region may affect mRNA stability, with this potentially influencing gene expression.

The factors controlling wool fibre curvature remains elusive, with various factors suggested, such as the relative abundance and distribution of orthocortical and paracortical cells [30], the relative length of these cells [31], or the structural arrangement of keratin intermediate filaments (KIFs) [32]. Given that HGT-*KRTAPs* are preferentially expressed in orthocortical cells [33], then variation in *KRTAP19-5* expression may affect the abundance and proportion of orthocortical and paracortical cells. Structural alterations in the KAP19-5 protein could also affect interactions with the KIFs and cross-linking processes in the fibre matrix, potentially altering the structural arrangement of KIFs and thus fibre properties like curvature.

While the mechanism(s) by which an individual KAP can affect fibre curvature remains uncertain, research has revealed connections between fibre curvature and the abundance of HGT-KAP proteins. For example, in Merino wool, a decrease in HGT-KAPs has been linked to the felting lustre mutation [34], and differences in the abundance of HGT-KAP proteins in wool fibres have been observed between wild-type and crimp mutant-type twins in both the Merino and Romney breeds [35]. These findings suggest that the levels of HGT-KAPs may indeed play a role in influencing fibre curvature. It has been suggested that there is a relationship between fibre curvature and fibre diameter, and that as fibre diameter increases staple crimp (reflecting changes in curvature) tends to decrease. Sumner [36] suggests that there are breed and selection lines that defy this generalization, but that the trait seems to be ‘due to the effect of a small number of genes’.

Plowman et al. [37] reported that the Merino wool fibres (fine wool) had a higher content of certain HGT-KAP proteins compared to the wool from Bordaleiro (medium wool) and Churro (coarse wool) sheep, and suggesting that the content of HGT-KAPs may decrease with larger fibres. If this holds true for KAP19-5, the content of KAP19-5 would likely be lower in the heterotypic hair fibres, with this possibly explaining why the effect on curvature was only observed in the fine wool fibres from the Chinese Tan sheep. Further research is certainly warranted to validate this observation.

The variant associated with decreased MFC (variant *B*), exhibited different frequencies in the two groups of sheep investigated in this study. Among the sheep of differing breed, variant *B* occurred at a frequency of 41.9%, but in the Chinese Tan sheep, this variant was detected at a much low frequency of 18.5%. This lower frequency observed in the Tan sheep may align with the high degree of ‘crimpiness’ that is characteristic of Tan sheep wool [16]. The identification of this variant may also present an opportunity to optimize wool curvature through selective breeding, with the removal of *B* from the population potentially increasing crimp.

The identification of seven *KRTAP19* genes within the human KAP19 family [15] leads us to hypothesize that there are of multiple *KRTAP19* genes in sheep as well. However, as other *KRTAP19* genes have not yet been identified in sheep, and need to be, their impact on wool traits remains uncertain. This makes it challenging to ascertain whether the association observed for *KRTAP19-5* is because of this specific gene, or is the result of linkage with hypothetically nearby, yet un-characterised *KRTAP19* genes. Further identification and investigation of these genes would provide valuable information in this respect. The *KRTAP19-n* genes are likely flanked by *KRTAP36-2* [13] and *KRTAP15-1* [37]. However, variation in *KRTAP36-2* was found to affect wool yield [13], while variation in *KRTAP15-1* was associated with wool yield and FDSD [38]. These findings suggest that the association detected for *KRTAP19-5* is less likely due to the influence of closely positioned genes from other KAP families.

## 5. Conclusions

This study identified a previously unknown KAP gene in sheep and reports variation in this gene. These findings add to our knowledge of wool protein genetics. The observed variation in *KRT19-5* was associated with wool mean fibre curvature. Further research is needed to confirm and validate these findings and also to further explore the relationship between *KRTAP19-5* and other wool traits, especially given the small population of sheep studied in the association part of this study, and the limited number of wool traits measured. These findings may be useful for the development of gene markers and thus enable the identification and selection of sheep for either increased or decreased fibre curvature. It should be part of an ongoing broader initiative to investigate the effect of wool protein genes on fibre traits.

## Figures and Tables

**Table 1:** Association of *KRTAP19-5* variants with wool traits in fine wool fibres of Chinese Tan sheep.

Trait ^1^	Variant ^2^	Mean ± SE ^3^		*p* Value
		Absent	Present	
MFD (µm)	*A*	16.7 ± 0.22	16.7 ± 0.20	0.707
	*B*	16.7 ± 0.19	16.7 ± 0.23	0.968
	*C*	16.6 ± 0.21	16.7 ± 0.20	0.729
	*D*	16.7 ± 0.18	16.6 ± 0.29	0.521
	*F*	16.7 ± 0.18	17.0 ± 0.30	0.250
FDSD (µm)	*A*	4.2 ± 0.16	4.2 ± 0.14	0.711
	*B*	4.2 ± 0.14	4.2 ± 0.17	0.823
	*C*	4.1 ± 0.15	4.2 ± 0.14	0.481
	*D*	4.2 ± 1.13	4.1 ± 0.21	0.421
	*F*	4.2 ± 0.13	4.3 ± 0.21	0.394
CVFD (%)	*A*	25.0 ± 0.73	24.8 ± 0.66	0.754
	*B*	24.9 ± 0.63	24.9 ± 0.78	0.999
	*C*	24.6 ± 0.70	25.1 ± 0.66	0.486
	*D*	25.0 ± 0.59	24.3 ± 0.96	0.421
	*F*	24.8 ± 0.59	25.3 ± 0.99	0.606
MFC (°/mm)	*A*	63.3 ± 1.37	65.1 ± 1.24	0.218
	*B*	**65.3 ± 1.19**	**62.3 ± 1.45**	**0.039**
	*C*	64.6 ± 1.33	64.1 ± 1.24	0.689
	*D*	64.2 ± 1.12	63.6 ± 1.83	0.643
	*F*	64.2 ± 1.11	65.1 ± 1.87	0.585

^1^ MFD—mean fibre diameter; FDSD—fibre diameter standard deviation; CVFD—coefficient of variation of fibre diameter; MFC—mean fibre curvature. ^2^ Among the 225 sheep (excluding those carrying the rare variant *E*), variant *A* was absent in 90 and present in 135 animals; variant *B* was absent in 147 and present in 78 animals; variant *C* was absent in 113 and present in 112 animals; variant *D* was absent in 185 and present in 40 animals; and variant *F* was absent in 187 and present in 38 animals. ^3^ Predicted means and standard errors derived from GLMs, with *p* < 0.05 being presented in bold.

## Data Availability

The original contributions presented in the study are included in the article, further inquiries can be directed to the corresponding author.

## Supplementary Materials

The following supporting information can be downloaded at: https://www.mdpi.com/article/10.3390/ani14152155/s1, Table S1: ESTs showing high similarity to ovine *KRTAP19-5* sequence ENSOARG00020032146.
