# Supplementary material for: Exploring Variation in Ovine KRTAP19-5 and Its Effect on Fine Wool Fibre Curvature in Chinese Tan Sheep
Source: Animals (Basel). 2024 Jul 24;14(15):2155. doi: 10.3390/ani14152155 (PMC11311106; doi:10.3390/ani14152155)
Supplement: Supplementary file 1 [file animals-14-02155-s001.zip › animals-3080640-supplementary.pdf]

Table S1. ESTs showing high similarity to ovine KRTAP19-5 sequence ENSOARG00020032146

Description	Max Score	Total Score	Query Cover	E value	Percentage identity	Acc. Len	Target Accession Number
001125OFSA014699HT OFSA Ovis aries cDNA 5', mRNA sequence	394	394	100%	1.00E-106	98.65	321	GO750511.1
030729OSCA3015038HT OSCA Ovis aries cDNA, mRNA sequence	394	394	100%	1.00E-106	98.65	528	EE847304.1
010914OSAB054065HT OSAB Ovis aries cDNA, mRNA sequence	388	388	100%	7.00E-105	98.2	524	EE845499.1
et5-55.z1 et adult sheep skin library Ovis aries cDNA, mRNA sequence	383	383	100%	3.00E-103	97.75	341	CF116192.1
